# Multimorbidity associated with functional independence among community-dwelling older people: a cross-sectional study in Southern China

**DOI:** 10.1186/s12955-017-0635-7

**Published:** 2017-04-17

**Authors:** Xiao-Xiao Wang, Wei-Quan Lin, Xu-Jia Chen, Ying-Yu Lin, Ling-Ling Huang, Sheng-Chao Zhang, Pei-Xi Wang

**Affiliations:** 1 Institute of Public Health, School of Nursing, Henan University, Kaifeng, 475004 China; 2 Guangzhou Center for Disease Control and Prevention, Guangzhou, 510440 China; 3 Community health service management center, Luohu hospital group, Shenzhen, 518007 China; 4 Baoan Central Hospital of Shenzhen, Shenzhen, 518102 China; 5 Department of Preventive Medicine, School of Public Health, Guangzhou Medical University, Guangzhou, 510182 China

**Keywords:** Multimorbidity, Chronic diseases, Functional independence, Older adults, Community-dwelling, China

## Abstract

**Background:**

Multimorbidity, the coexistence of two or more chronic diseases, is common in older adults. And it may lead to many adverse health outcomes, such as disability. However, data on multimorbidity and its relationship with functional independence are scarce in Asian countries. Therefore, this study aims to investigate the relationship between multimorbidity and functional status among older people in China.

**Methods:**

Based on a cross-sectional survey, the information regarding 2705 older adults, who were of at least 60 years of age, was collected through interviews and analyzed. To assess functional status, we used the Functional Independence Measure (FIM). Exploratory factor analysis was performed to assess correlations among chronic diseases. Several logistic regression models were run in the study.

**Results:**

The presence of two or more chronic conditions and the number of multimorbidity group overlaps were independent risk factors for the loss of functional independence in older adults. Hypertension and chronic pain, emerged as the most prevalent multimorbidity pair, was significantly associated with functional independence (OR = 1.64, 95% CI = 1.25–2.16), followed by the co-occurrence of hypertension and heart diseases with a lower prevalence but a higher OR compared with the former pair (OR = 1.72, 95% CI = 1.15–2.58). Of the five multimorbidity groups used for factor analysis, the bones and pain group (OR = 1.47, 95% CI = 1.23–1.77) and the cardiometabolic group (OR = 1.34, 95% CI = 1.13–1.59) were both found to be significantly correlated with lower functional independence.

**Conclusions:**

Multimorbidity was common among older people in Southern China. Studying the relationship between multimorbidity and functional status could be useful to find potential correlations among chronic diseases. Additionally, it may also be meaningful to identify multimorbidity combinations, posing an increased risk of loss of functional independence, and further improve functional status in older adults with comorbidities.

## Background

Multimorbidity, usually defined as the co-existence of two or more chronic health conditions in one person [1, 2], is becoming the norm rather than the exception in older adults [3, 4]. As populations age, the prevalence of multimorbidity will rise along with the absolute increase in the number of patients with chronic conditions. Statistics show that, by 2050, the world population will include two billion people over the age of 60, around 85% of who will be living in today’s developing countries [2]. A recent systematic review showed that the prevalence of multimorbidity is greater than 60% worldwide, and is probably greater than 80% among those aged ≥85 years [5]. It varies widely, owing to the lack of consensus on multimorbidity measurements, such as the number and types of chronic conditions. Currently, multimorbidity has been a worldwide public health tissue and is associated with many adverse health outcomes, such as mortality, disability, poor quality of life, hospitalizations and use of health care resources and expenditures [6–9].

The International Classification of Functioning, Disability and Health (ICF) defined functional status as activity and participation, including communication, mobility, interpersonal interactions, and self-care [10, 11]. And for community-dwelling people, the key problem tends to be whether they can take care of themselves or survive in the community. Although considering the natural loss of function as age increases, some specific diseases, such as arthritis and arthrosis [12], stroke and diabetes [13], and the total number of chronic diseases [14] were associated with the lower functional status. Moreover, a few previous studies have shown that comorbidity is a strong risk factor for disability in itself [15], and may also have an additive or synergistic effect on disability [16].

To our knowledge, numerous epidemiologic studies on multimorbidity have been conducted in most countries. Multimorbidity may be used to identify older adults with increased vulnerability toward adverse outcomes [17]. However, data on multimorbidity, and particularly its relationship with functional independence, are scarce in Asian countries [4]. In China, a country with large population, the aging of population as well as the absolute increases of number of chronic diseases may result in the increase in the likelihood of multimorbidity [18]. Moreover, the information about the health of elderly individuals is greatly needed.

Therefore, regarding the increasing importance of multimorbidity and the lack of detailed studies regarding the relationship between multimorbidity and functional status, we conducted a cross-sectional analysis in older adults aged ≥60 years of age in Southern China. Therefore, the study aimed to (1) confirm the effect of the number of chronic diseases on functional status, (2) assess the prevalence of each chronic disease and multimorbidity conditions and identify meaningful multimorbidity patterns, and (3) explore how those multimorbidity patterns affect the functional independence of older adults in the community.

## Methods

### Study design and populations

This study was based on a cross-sectional community health survey in the Shenzhen city of Guangdong province in Southern China. The samples in this survey were selected using a multistage and stratified random sampling method, consisting of family members drawn from five percent of the total households in this municipality. In the present study, we used the available information from people aged ≥60 years of age. Of 2919 participants, 214 (7.33%) subjects were excluded due to incomplete or inconsistent data from questionnaires. Finally, a total of 2705 older adults were included in our analysis.

### Measurements

#### Participants’ characteristics

The variables used for the analysis included age, sex, marital status, education level, employment status, individual economic conditions and body mass index (BMI). We used dummy variables for age group categorized as 60–69, 70–79 and ≥80 years old. Marital status was divided into two categories: currently married and single (including unmarried, divorced or widowed). As for the highest level of education participants had attained, responses were grouped into three categories as follows: primary school or lower, middle school, high school or above. Employment status was categorized into employed and unemployed. In this study, the definition of unemployed included all subjects who were without work [19]. Individual economic condition was divided into two groups based on the question stem “what do you think of your financial situation…”. According to the Chinese BMI reference [20], the BMI was calculated as weight (kg) divided by the square of height (m^2^), and all participants were categorized into four groups.

### Chronic diseases measure

The presence of chronic diseases was determined via self-report, as a yes/no response to the question stem, “has a doctor ever diagnosed that you had…” [21]. The 17 diseases investigated in this study included: hypertension, chronic pain, diabetes mellitus, hyperlipidemia, bone diseases, gastroenteritis, heart disease, gout, peripheral vascular disease, chronic kidney disease, spleen and gallbladder diseases, pulmonary disease, stroke, cancer, multiple sclerosis, dementia and mental disorder. Multimorbidity was defined as the presence of two or more of the 17 chronic diseases in an individual.

### Functional independence measure

Functional status was assessed by the Functional Independence Measure (FIM) in this paper. The FIM score is defined as the level of assistance required for an individual to perform activities of daily living indicating the burden of caring for them [22]. The scale includes 18 items, of which 13 items are physical domains based on the Barthel Index and 5 items are cognition items. Each item is scored from 1 to 7, based on the level of independence, where 1 represents total assistance and 7 indicates complete independence [23]. Possible scores range from 18 to 126, with higher scores indicating more independence. To our knowledge, the 18-item FIM instrument has been found to be reliable and well validated [24, 25]. And the Cronbach’ Alpha of FIM via Reliability Analysis was 0.928 in this paper. Considering the non-normal distribution of the data and the high percentage of well-functioning participants, we dichotomized functional status, the dependent variable, into “completely independent” (=126 scores) and “not completely independent” (<126 scores) according to their FIM scores. Finally, all participants were categorized into two groups: those completely independent, and those needing some help or assistance with activities of daily living.

### Statistical analysis

Descriptive statistics were calculated for all measures. Means and standard deviations (SD) were presented for continuous variables, while frequency and percentage were used for categorical variables. To analyze the relationship between multimorbidity and functional independence, several logistic regression models were run.

First, we evaluated the association between the number of chronic diseases and functional independence after adjusting for demographic characteristics and other possible confounding factors. Subsequently, based on the results-of this preliminary analysis, multimorbidity and its relationship with functional independence were explored, in depth.

The spectrum of 17 chronic diseases was displayed by calculating the prevalence of each disease individually. We chose prevalent chronic medical conditions with a prevalence >10%, and analyzed their correlations using contingency tables to generate the most prevalent multimorbidity pairs (the combination of two chronic diseases) and conducted a logistic regression analysis for each of the top 10 multimorbidity pairs by adjusting for other confounding factors.

To analyze how health conditions are grouped together, we used an exploratory factor analysis with principal components using a tetrachoric correlation matrix [26], which allows that a chronic condition can load onto several factors or multimorbidity groups. The Kaiser-Meyer-Olkin measure of sampling and the Bartlett’s test of sphericity were performed to investigate sampling adequacy for conducting factor analysis. The rotation method was the oblique direct oblimin, which allows factors to be associated with each other. Subjects were assigned to a group if they were diagnosed with at least 2 of the diseases included in the group. Multimorbidity groups overlap when a person is assigned to two or more groups.

Then, to explore the impact of multimorbidity groups in functional independence, we conducted a logistic regression analysis adjusting for confounding factors. Additionally, another logistic regression model was performed to assess how overlapping groups affected functional status.

All statistical analyses were conducted using the Statistical Package for the Social Sciences (SPSS) version 13.0 (SPSS Inc., Chicago, IL, USA). Two-tailed analyses were performed with the level of significance set at *P* <0.05.

## Results

Of the total 2705 older adults, aged 60 years and above, with an average age of (69.24 ± 7.58) years, 58.2% were women and a majority of participants were married. Among them, more than half received an education of primary school or lower, with a significant difference between men (40.0%) and women (60.3%), and only a few of the elderly people still were employed. Moreover, 45.4% of individuals reported they thought their economic conditions were not good. Almost half of them had a normal BMI. The average number of chronic conditions was (1.68 ± 1.60). A multimorbidity status (≥2 chronic diseases) was reported in 45.5% of the study sample. Sex-related differences were found to be significant in our study and women had more multimorbidity compared with men (48.6% and 41.1%, respectively). The FIM mean score was 119.66 ± 12.79, and more than half of the elderly people (62.3%) were not completely independent. The socio-demographic and health characteristics of the individuals in the sample set are detailed in Table 1.

**Table 1:** Socio-demographic and health characteristics of study participants

		Sample (*N* = 2705)
Age, mean (SD)		69.24 (7.58)
Age group, years, *n* (%)	60 ~ 69	1644 (60.8)
	70 ~ 79	736 (27.2)
	80~	325 (12.0)
Gender, *n* (%)	Male	1131 (41.8)
	Female	1574 (58.2)
Marital status, *n* (%)	Married	2074 (76.7)
	Single	631 (23.3)
Education level, *n* (%)	Primary school or lower	1401 (51.8)
	Middle school	597 (22.1)
	High school or above	707 (26.1)
Employment status, *n* (%)	Employed	133 (4.9)
	Unemployed	2572 (95.1)
Individual economic condition, *n* (%)	Good	1477 (54.6)
	Not good	1228 (45.4)
Body mass index, mean (SD)		23.90 (3.90)
Body mass index, kg/m^2^, *n* (%)	<18.5	124 (4.6)
	18.5 ~ 23.9	1317 (48.7)
	24 ~ 27.9	981 (36.3)
	≥28	283 (10.5)
Number of chronic diseases, *n* (%)	0	663 (24.5)
	1	812 (30.0)
	≥2	1230 (45.5)
FIM score, mean (SD)		119.66 (12.79)
FIM category, n (%)	Complete independent	1021 (37.7)
	Not complete independent	1684 (62.3)

*Single* unmarried, divorced, windowed, *FIM* Functional independence measure

Table 2 shows univariate and multivariate logistic regression models analyzing functional independence (dependent variable) by sample characteristics. After adjusting for possible confounding factors, which were associated with higher functional independence, multimorbidity was strongly associated with lower functional status (OR = 2.06, 95% CI = 1.68–2.53), followed by advanced age group (≥80 years old) (OR = 3.62, 95% CI = 2.59–5.09).

**Table 2:** Logistic regression models analyzing the relationship between functional independence and sample characteristics

		Crude OR (95% CI)	Adjusted^a^ OR (95% CI)
Age group, years	70 ~ 79	1.46 (1.22–1.75)^**^	1.40 (1.15–1.69)^**^
	80~	4.36 (3.17–5.99)^**^	3.62 (2.59–5.09)^**^
Gender	Female	1.31 (1.12–1.54)^**^	1.12 (0.94–1.33)
Marital status	Single	1.60 (1.32–1.94)^**^	1.05 (0.85–1.30)
Education level	Middle school	0.56 (0.46–0.69)^**^	0.65 (0.53–0.80)^*^
	High school or above	0.49 (0.41–0.59)^**^	0.54 (0.45–0.66)^**^
Employment status	Unemployed	1.72 (1.21–2.44)^**^	1.34 (0.93–1.93)
Individual economic	Good	0.77 (0.66–0.90)^**^	0.75 (0.64–0.89)^**^
Body mass index,kg/m^2^	<18.5	1.16 (0.79–1.71)	0.94 (0.63–1.42)
	24 ~ 27.9	0.91 (0.77–1.08)	0.92 (0.77–1.10)
	≥28	1.07 (0.82–1.40)	0.95 (0.71–1.25)
No. of chronic diseases	1	1.32 (1.08–1.63)^**^	1.22 (0.98–1.51)
	≥2	2.21 (1.82–2.69)^**^	2.06 (1.68–2.53)^**^

^a^Multivariate logistic regression models adjusted by age, gender, marital status, education level, employment status, individual economic conditions, body mass index and self-reported chronic diseases. *No.* Number, *OR* Odds ratio, *CI* Confidence interval. ^*^ *P* < 0.05, ^**^ *P* < 0.01

Table 3 displays the rank prevalence of 17 self-reported chronic diseases and the most prevalent related pairs of these diseases with a prevalence >10% in the sample population. The most common chronic disease in the population was hypertension (44.9%), followed by chronic pain (27.0%). In addition, diabetes mellitus and hyperlipidemia had a prevalence of 16.8% and 13.9%, respectively. Furthermore, significant differences based on sex were found in some diseases. For example, women vs. men reported hypertension (46.6% vs. 42.4%), diabetes mellitus (18.1% vs. 15%), chronic pain (30.4% vs. 22.4%) and bone diseases (14.9% vs. 9.8%), all of which were more prevalent in women. Conversely, stroke was more prevalent in men (3.8% vs. 2.3%).

**Table 3:** The rank prevalence of 17 self-reported chronic diseases and most prevalent related pairs of chronic diseases with a percentage >10% in the sample population

Type of single disease	Sample, *n* (%)	95% CI	Rank	Pairs of diseases^a^	Sample, *n* (%)	95% CI
Hypertension	1214 (44.9)	42.9%, 46.9%	1	HT + CP	359 (13.3)	11.9%, 14.7%
Chronic pain	731 (27.0)	25.2%, 28.8%	2	HT + DM	273 (10.1)	8.9%, 11.3%
Diabetes mellitus	455 (16.8)	15.4%, 18.2%	3	HT + HL	230 (8.5)	7.5%, 9.5%
Hyperlipidemia	375 (13.9)	12.5%, 15.3%	4	HT + BD	200 (7.4)	6.4%, 8.4%
Bone diseases	346 (12.8)	11.6%, 14.0%	5	CP + BD	166 (6.1)	5.1%, 7.1%
Gastroenteritis	320 (11.8)	10.6%, 13.0%	6	HT + HD	164 (6.1)	5.1%, 7.1%
Heart disease	283 (10.5)	9.3%, 11.7%	7	CP + HL	148 (5.5)	4.7%, 6.3%
Gout	194 (7.2)	6.2%, 8.2%	8	CP + GE	146 (5.4)	4.6%, 6.2%
Peripheral vascular disease	157 (5.8)	5.0%, 6.6%	9	CP + HD	118 (4.4)	3.6%, 5.2%
Chronic kidney disease	118 (4.4)	3.6%, 5.2%	10	DM + HL	107 (4.0)	3.2%, 4.8%
Spleen and gallbladder diseases	110 (4.1)	3.3%, 4.9%	11	HL + HD	82 (3.0)	2.4%, 3.6%
Pulmonary disease	102 (3.8)	3.0%, 4.6%	12	HL + GE	79 (2.9)	2.3%, 3.5%
Stroke	79 (2.9)	2.3%, 3.5%	13	BD + GE	77 (2.8)	2.2%, 3.4%
Cancer	29 (1.1)	0.7%, 1.5%	14	HL + BD	76 (2.8)	2.2%, 3.4%
Multiple sclerosis	19 (0.7)	0.3%, 1.1%	15	DM + HD	62 (2.3)	1.7%, 2.9%
Dementia	18 (0.7)	0.3%, 1.1%	16	GE + HD	58 (2.1)	1.5%, 2.7%
Mental disorder	6 (0.2)	0.03%, 0.4%	17	BD + HD	53 (2.0)	1.4%, 2.6%

^a^All pairs have significantly correlation (*P* < 0.05). *CI* Confidence interval, *HT* Hypertension, *CP* Chronic pain, *DM* Diabetes mellitus, *HL* Hyperlipidemia, *BD* Bone diseases, *HD* Heart disease, *GE* Gastroenteritis

Table 4 presents the logistic regression models analyzing the relationships between functional independence and the 10 most common multimorbidity pairs. After adjusting for other confounding factors, there were two prevalent multimorbidity pairs of chronic conditions significantly associated with functional independence. The most prevalent pair was the combination of hypertension and chronic pain (OR = 1.64, 95% CI = 1.25–2.16), followed by hypertension and heart diseases, which had lower prevalence but the highest OR (OR = 1.72, 95% CI = 1.15–2.58).

**Table 4:** Logistic regression models analyzing the relationships of functional independence and most prevalent multimorbidity pairs

Top10 pairs of diseases		Crude OR (95% CI)	Adjusted^a^ OR (95% CI)
Hypertension	Chronic pain	2.18 (1.68–2.82)^**^	1.64 (1.25–2.16)^**^
Hypertension	Diabetes mellitus	1.41 (1.08–1.85)^*^	1.28 (0.96–1.71)
Hypertension	Hyperlipidemia	1.37 (1.02–1.82)^*^	1.16 (0.85–1.60)
Hypertension	Bone diseases	2.01 (1.44–2.81)^**^	1.40 (0.98–2.01)
Chronic pain	Bone diseases	1.53 (1.08–2.16)^*^	1.10 (0.76–1.59)
Hypertension	Heart disease	2.17 (1.49–3.16)^**^	1.72 (1.15–2.58)^**^
Chronic pain	Hyperlipidemia	1.62 (1.12–2.35)^*^	1.28 (0.86–1.92)
Chronic pain	Gastroenteritis	1.77 (1.22–2.59)^**^	1.44 (0.96–2.16)
Chronic pain	Heart disease	2.21 (1.42–3.44)^**^	1.54 (0.96–2.48)
Diabetes mellitus	Hyperlipidemia	1.37 (1.02–1.82)^*^	0.96 (0.62–1.48)

^a^Models per group, adjusted for age, gender, marital status, education level, employment, individual economic conditions, body mass index and all the other single diseases. *OR* Odds ratio, *CI* Confidence interval. ^*^ *P* < 0.05, ^**^ *P* < 0.01

**Fig. 1:**
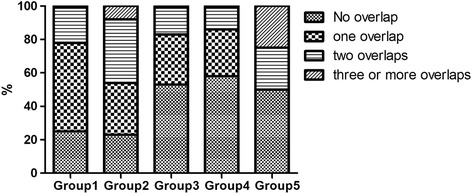
Proportion of overlap between five multimorbidity groups

The results of the factor analysis and the relationship between factors and functional status are presented in Table 5. Sampling adequacy for factor analysis was confirmed by KMO = 0.699 and Bartlett’s test of sphericity (*P* < 0.001). Finally, five groups emerged, with an explained total variance of 40.42%. The first group included spleen and gallbladder diseases, chronic kidney disease, gastroenteritis, heart disease, peripheral vascular disease and hyperlipidemia (SGD-CKD-GE and PVD group). The second group included dementia, mental disorders, multiple sclerosis, and stroke (neuropsychiatric and stroke group). The third group included bone diseases, chronic pain and gout (bones and pain group). The fourth group made up of hypertension, diabetes mellitus and hyperlipidemia (cardio metabolic group). Lastly, the fifth group included pulmonary disease and cancer (lung and cancer group). The cardiometabolic group had the highest prevalence (16.3%), followed by the bones and pain group (13.8%). In the logistic regression models, the bones and pain group (OR = 1.47, 95% CI = 1.23–1.77) and the cardiometabolic group (OR = 1.34, 95% CI = 1.13–1.59) were all significantly associated with lower functional independence, even after controlling for other confounders. Totally, 29.4% of participants were assigned to at least one multimorbidity group. The overlap of multimorbidity patterns is presented in Fig. 1. Most of the subjects, included in the SGD-CKD-GE and PVD group, as well as the neuropsychiatric and stroke group, also belonged to other groups (one or more overlapping multimorbidity patterns). Another logistic regression model, adjusted for confounding factors, found that compared to those with no overlap or only belonging to one of multimorbidity groups, those in whom-two groups of multimorbidity patterns overlap showed an OR of 1.51 (95% CI = 1.10–2.09) and when three or more groups overlap there was an OR of 2.07 (95% CI = 1.11–3.84).

**Table 5:** Loadings of factors with eigenvalue >1 and logistic regression analysis between functional independence and multimorbidity groups

	Factor loadings				
	SGD-CKD-GE and PVD group	Neuropsychiatric-stroke group	Bones and pain group	Cardiometabolic group	Lung and cancer group
*Factor analysis*					
Hypertension				0.64	
Diabetes mellitus				0.63	
Hyperlipidemia	0.50			0.44	
PVD	0.45				
Heart disease	0.44				
CKD	0.48				
SGD	0.60				
Gastroenteritis	0.45				
Chronic pain			0.63		
Bone diseases			0.68		
Gout			0.38		
Pulmonary disease					0.68
Cancer					0.65
Dementia		0.72			
Mental disorder		0.70			
Stroke		0.44			
Multiple sclerosis		0.52			
Prevalence, n (%)	291 (10.8)	13 (0.5)	372 (13.8)	442 (16.3)	4 (0.1)
*Regression analysis*					
Crude OR (95% CI)	1.48(1.14–1.93)^**^	7.32(0.95–56.38)	1.69(1.33–2.16)^**^	1.43(1.15–1.78)^**^	1.82(0.19–17.52)
Adjusted OR (95% CI)	1.29(0.96–1.74)	3.97(0.50–31.72)	1.41(1.09–1.84)^**^	1.31(1.03–1.66)^*^	1.61(0.15–16.72)

Factor loadings <0.35 have been omitted. *SGD* Spleen and gallbladder diseases, *GE* Gastroenteritis, *PVD* Peripheral vascular disease, *CKD* Chronic kidney disease. ^a^Models per group, adjusted for age, gender, marital status, education level, employment, individual economic conditions, body mass index and all the other factors. *OR* Odds ratio, *CI* Confidence interval. ^*^ *P* < 0.05, ^**^ *P* < 0.01

## Discussion

### Main findings

Within a sample of Chinese older adults, we found that the number of chronic conditions (≥2 diseases) was independently associated with lower functional independence. Based on this, we wanted to understand how different multimorbidity patterns affect functional independence. We found that hypertension and chronic pain account for the biggest loss in functional independence, whether alone or co-occuring. Their associations with functional independence persisted, even after adjusting for confounders. Of the five multimorbidity groups, the bones and pain group as well as the cardiovascular and metabolic groups were identified as having a significant risk of reduced functional independence. Interestingly, we detected that the number of overlap multimorbidity groups was also greatly related to functional status in individuals.

### Comparison with previous studies

In our study, the great majority of older adults suffered from chronic diseases and 45.5% reported the presence of two or more chronic conditions. It fits well with the range of the overall prevalence of multimorbidity from a systematic review of 9 published studies in China (6.4%–76.5%) [27]. A study with a large sample conducted in Southern China showed that the prevalence of multimorbidity among the elderly people was 47.5% [18], consistent with our results. Multimorbidity is a common worldwide phenomenon among the elderly population, and China is no exception. Additionally, gender differences were significant whether in multimorbidity or single diseases. Women had a higher prevalence of multimorbidity, and were more prone to suffer from pain [28], bone diseases and hypertension [10] than men. Women had a longer life expectancy [29], and thus, they were affected more often by chronic disease than men. On the other hand, the low hormone levels characteristic of menopause can accelerate bone loss [30] and atherosclerosis progression [31], which could partially explain the sex-specific distribution of diseases. However, sex was not found to be independently associated with functional independence in our analysis. A review of the studies also found that after adjusting for socioeconomic, health, and social relations indicators, incidence of functional disability was similar between genders [32]. The effect of sex on functional status remains to be further studied.

The first logistic regression model we ran showed that the number of multiple chronic conditions was independently associated with functional independence. Subjects with two or more chronic diseases had a higher risk of losing functional independence, as found in the previous literatures [14, 33]. A community-based follow-up study also revealed that multimorbidity increased the odds of being in the “With disability” group at follow-up [34]. Age, especially senile age, was another independent risk factor, consistent with previous studies [12, 35]. With the aging process, the older adults would inevitably experience loss of strength, osteoporosis or other degenerative changes in addition to loss of balance that may have a greater impact on functional independence. Both higher education level and better economic conditions were associated with higher functional independence, consistent with other studies [33, 36], which could be elucidated by stronger health awareness, higher income and better access to prevention, treatment and rehabilitation. Under this premise, how different types of multimorbidity affect functional independence was the focus of our analysis.

Hypertension was found to be the single most prevalent disease and was included in both of the multimorbidity pairs with statistical significance related to functional status, specifically, heart diseases and chronic pain. Compared with single diseases, multimorbidity pairs had a higher risk of low functional independence. In fact, hypertension, which is very common in China [37], can lead to left ventricular hypertrophy and progression of atherosclerosis, which are leading causes of heart failure and ischemic heart disease [38, 39]. Chamberlain et al [40] found that hypertension was one of the most prevalent diseases in heart failure patients. Previous studies have also demonstrated that the presence of chronic pain was associated with significantly increased odds of hypertension [41].

Regarding chronic diseases clusters, we detected five multimorbidity groups using factor analysis, as performed by other authors exploring multimorbidity patterns [10, 26, 42, 43]. The first group, the SGD-CKD-GE and PVD group had a high prevalence of 10.8% but had no statistically significant association with functional status after the adjustment. The second group, including dementia, mental disorders, multiple sclerosis and stroke, had a lower prevalence (0.5%) than the SGD-CKD-GE and PVD group. Although the OR of the effect on functional dependence was the highest, the significance was not statistically significant, which may be attributable to the dichotomization of the dependent variable. The third group, which included bone diseases, gout and chronic pain, was significantly associated with functional independence. Bone diseases such as osteoarthritis [32, 35], arthrosis and osteoporosis [36] had a great effect on functional disability. Komatsu et al [44] found that muscle, bone and joint pain were independently associated with lower ADL level, which was accordant with our findings. Meanwhile, chronic pain, a prevalent condition in elderly patients, was associated with greater reported difficulty in performing certain essential self-management activities [45]. Importantly, Connolly et al [46] noted that, after age, pain was one of the strongest factors associated with difficulty in ADL/IADL combined and ADL alone. However, it was not included in the previous published article by Marventano S et al [10]. Given the strong influence of chronic pain on difficulty with functional independence, early interventions for pain reduction, control, and self-management should be performed and future studies on multimorbidity should consider inclusion of chronic pain. The cardio metabolic group is also prominent with the highest prevalence among older people, followed by the bones and pain group. In China, a large investigation conducted by Wang et al [47] showed high prevalence of hypertension, diabetes and hyperlipidemia, proving them to be common disease in the elderly population. As we all know, hypertension, diabetes mellitus and hyperlipidemia are all established risk factors for cardiovascular diseases [48], being part of the so-called “metabolic syndrome” [49]. The lung and cancer group had the lowest prevalence in our population. The cancer tended to affect the respiratory tracts as the most frequent sites [10].

Considering the presence of multiple chronic diseases with various complex interactions, subjects with multimorbidity had greater difficulty in performing daily activities and had greater need for care. More importantly, we found an interesting dose-response association. Specifically, the greater the overlap between multimorbidity groups, the greater was the impact on functional independence in older adults.

However, this study has some limitations. First, this is a cross-sectional study. Further in-depth studies with a longitudinal follow-up data are warranted to examine the cause-effect relationship. Second, the lack of inclusion of other chronic conditions may underestimate the prevalence of multimorbidity. This could be the reason for the relatively low prevalence of multimorbidity. Finally, information about the severity of disease should be taken into consideration in future studies.

## Conclusions

With the study samples drawn from the world’s largest developing country, we have provided information on the prevalence of multimorbidity and different patterns of multimorbidity, as well as their relationships with functional independence in older adults. Findings from this study have significant implications for identifying multimorbid patients at risk of losing functional independence, and developing interventions aimed at these individuals. This will help them maintain functional level and help to prevent disability. Future research should further explore the distribution of multimorbidity patterns in different regions, as well as the relationship of multimorbidity and functional independence in a prospective cohort study.

## Abbreviations

95% CI: 95% confidence intervals
ADL: Activities of daily life
BMI: Body mass index
CKD: Chronic kidney disease
FIM: Functional independence measure
GE: Gastroenteritis
IADL: Instrumental of activities of daily life
KMO: Kaiser-Meyer-Olkin measure
OR: Odds radios
PVD: Peripheral vascular disease
SGD: Spleen and gallbladder diseases
